# Photodynamic Therapy Review: Principles, Photosensitizers, Applications, and Future Directions

**DOI:** 10.3390/pharmaceutics13091332

**Published:** 2021-08-25

**Authors:** José H. Correia, José A. Rodrigues, Sara Pimenta, Tao Dong, Zhaochu Yang

**Affiliations:** 1 Chongqing Key Laboratory of Micro-Nano Systems and Smart Transduction, Collaborative Innovation Center on Micro-Nano Transduction and Intelligent Eco-Internet of Things, Chongqing Key Laboratory of Colleges and Universities on Micro-Nano Systems Technology and Smart Transducing, National Research Base of Intelligent Manufacturing Service, Chongqing Technology and Business University, Nan’an District, Chongqing 400067, China; higino.correia@dei.uminho.pt (J.H.C.); tao.dong@usn.no (T.D.); zhaochu.yang@ctbu.edu.cn (Z.Y.); 2 CMEMS-UMinho, Department of Industrial Electronics, University of Minho, 4800-058 Guimaraes, Portugal; sara.pimenta@dei.uminho.pt; 3 Department of Microsystems-IMS, Faculty of Technology, Natural Sciences and Maritime Sciences, University of South-Eastern Norway (USN), Postboks 235, 3603 Kongsberg, Norway

**Keywords:** PDT mechanisms, new photosensitizers, PDT tumor treatment, antimicrobial PDT, non-oncologic applications of PDT, PDT in medical devices

## Abstract

Photodynamic therapy (PDT) is a minimally invasive therapeutic modality that has gained great attention in the past years as a new therapy for cancer treatment. PDT uses photosensitizers that, after being excited by light at a specific wavelength, react with the molecular oxygen to create reactive oxygen species in the target tissue, resulting in cell death. Compared to conventional therapeutic modalities, PDT presents greater selectivity against tumor cells, due to the use of photosensitizers that are preferably localized in tumor lesions, and the precise light irradiation of these lesions. This paper presents a review of the principles, mechanisms, photosensitizers, and current applications of PDT. Moreover, the future path on the research of new photosensitizers with enhanced tumor selectivity, featuring the improvement of PDT effectiveness, has also been addressed. Finally, new applications of PDT have been covered.

## 1. Introduction

### 1.1. History of Photodynamic Therapy

Light has been used in the treatment of several diseases since antiquity [1]. The ancient civilizations, Egyptian, Indian, and Chinese, used the sunlight to treat various skin diseases, such as psoriasis, vitiligo, and skin cancer [2]. Herodotus, a famous Greek physician known as the father of heliotherapy, emphasized the importance of whole-body sun exposure for the restoration of health. In the 18th and 19th centuries, in France, sunlight was used in the treatment of several conditions, such as tuberculosis, rickets, scurvy, rheumatism, paralysis, edema, and muscle weakness [3]. At the beginning of the 20th century, the importance of light in the treatment of diseases was recognized, and the 1903 Nobel Prize in Physiology or Medicine was awarded to Niels Finsen for his contribution in this field. Finsen found that sunlight or light from a carbon arc lamp with a heat filter could be used to treat lupus vulgaris, a tubercular condition of the skin. This discovery marked the beginning of modern phototherapy [1,4,5].

Phototherapy describes the use of light in the treatment of a disease. However, photochemotherapy requires the administration of a photosensitizing agent, which is subsequently activated by light in the tissues, where the agent is localized. This form of therapy also dates back over 3000 years, when the Indians and Egyptians used psoralens from natural plants in the treatment of a variety of skin conditions [3,6].

The concept of cell death induced by the interaction of light and chemicals has been recognized for 100 years. In 1900, a German medical student, O. Raab, first reported cell death induced by the interaction of light with chemicals. While working with Professor H. von Tappeiner in Munich, he described the lethal effect that the combination of light and acridine red had on protozoa [3,4,6]. In subsequent experiments, Raab demonstrated that this lethal effect was greater than with acridine red alone, light alone, or acridine red exposed to light and then added the protozoan. He reported that toxicity occurred as a result of fluorescence caused by the transfer of energy from light to the chemical [6,7]. In the same year, the French neurologist J. Prime discovered that patients with epilepsy who were treated with orally administered eosin developed dermatitis in areas exposed to sunlight. Later, in 1903, H. von Tappeiner and A. Jesionek treated skin tumors with topical applications of eosin and white light [3,4,6]. In 1904, H. von Tappeiner and A. Jodlbauer identified that oxygen is an integral component in photosensitization reactions, and in 1907 they introduced the term “photodynamic action” to describe this phenomenon [1,2,3,6].

In 1841, H. Scherer first produced hematoporphyrin while investigating the nature of blood. However, its fluorescent properties were not described until 1867, and this substance was only named by hematoporphyrin in 1871. In 1911, W. Hausmann reported the effects of hematoporphyrin and light on protozoa and blood cells, describing skin reactions in a mice exposed to light after being administered with hematoporphyrin [3,4,6]. The first report of human photosensitization was in 1913, when F. Meyer-Betz injected himself with 200 mg of hematoporphyrin to determine if similar effects could be induced in humans. He described prolonged pain and swelling in light-exposed areas [2,3,6]. In 1960, R. Lipson and S. Schwartz initiated the concept of photodynamic therapy (PDT) at the Mayo Clinic, discovering the cancer diagnostic and therapeutic effects by injecting a hematoporphyrin derivative (HpD) [1]. In 1975, a significant breakthrough in PDT occurred, when T. Dougherty reported that administration of HpD and its activation with red light completely eradicated the growth of mammary tumor in mice. In the same year, J. F. Kelly proved the elimination of bladder carcinoma in mice, by activating HpD with light [4]. In 1976, another major event in the development of PDT occurred, when J. F. Kelly and M. E. Snell proceeded to the first human study of the effects of PDT in bladder cancer using HpD [3]. The use of this technique in the treatment of pathologies in the gastrointestinal tract was first performed in 1984 by J. S. McCaughan, who used PDT to treat patients with esophageal cancer. One year later, Y. Hayata used PDT to treat patients with gastric carcinoma [3,4]. Dougherty and his coworkers also purified HpD and produced Photofrin, which was the first photosensitizer molecule (PS) approved by the US Food and Drug Administration (FDA) in 1995 for cancer treatment [2]. Since then, PDT has continued to evolve and its clinical application was extended to other areas besides tumors treatment. Dr. M. Weber, known as the pioneer of modern laser therapy, also applies PDT to treat bacterial, viral, and parasitic diseases, named as antimicrobial PDT. The main advantage of this new approach is to combat multiresistant pathogens. Dr. Weber developed the Weberneedle^®^ technology, which allows to apply highly focused and efficient lasers of different wavelengths to intravenous, interstitial, and intra-articular irradiation [8,9].

### 1.2. Principles of PDT

**Figure 1:**
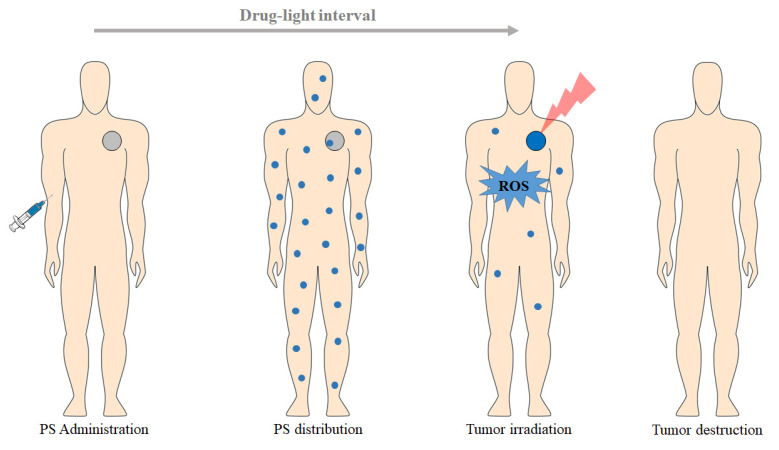
Representation of the clinical application of PDT protocol for the treatment of a solid and localized tumor (based on [12]).

PDT is based on the dynamic interaction between a PS, light with a specific wavelength, and molecular oxygen, promoting the selective destruction of the target tissue [10]. The PDT treatment consists in the administration of a PS (topically or intravenous), which selectively accumulates in the tumor tissue (during a drug–light interval), followed by subsequent exposition to an appropriate wavelength light (generally in the red spectral region, λ ≥ 600 nm; Figure 1) [11]. The PS itself does not react with biomolecules; however, illumination transfers energy from light to molecular oxygen, to generate reactive oxygen species (ROS), such as singlet oxygen (^1^O_2_), superoxide radical (O_2_^−•^), hydroxyl radical (HO^•^), and hydrogen peroxide (H_2_O_2_) [1]. These cytotoxic photoproducts start a cascade of biochemical events, which can induce damage and death of the target tissue [11].

## 2. Photodynamic Reaction

**Figure 2:**
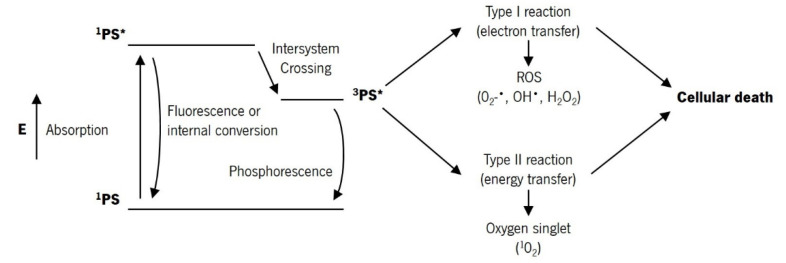
Modified Jablonski diagram of the PDT action mechanism (based on [13]).

PDT is a therapeutic modality based on the combination of three factors: PS, light with a specific wavelength, and the presence of molecular oxygen [2]. The photodynamic reaction begins with the absorption of light by the PS in the target tissue, which triggers a series of photochemical reactions that lead to the generation of ROS [10]. After light absorption, the PS is transformed from its ground state (singlet state, ^1^PS) to a short-lived, electronically excited singlet state (a few nanoseconds or less, ^1^PS*) [2,4]. This excited state is very unstable and can decay to the ground state, losing the excess of energy through light emission (fluorescence) or heat production (internal conversion) [13]. However, the singlet state can undergo intersystem crossing and progress to a more stable, long-lived, electronically excited state (triplet state, ^3^PS*), through spin conversion of the electron in the higher-energy orbital [13]. This excited state can decay to the ground state through light emission (phosphorescence) or undergo two kinds of reactions [4]. The triplet state has a longer lifetime (up to tens of microseconds), which allows sufficient time for direct transfer of energy to molecular oxygen (O_2_). This energy transfer step leads to the formation of singlet oxygen (^1^O_2_) and the fundamental state of the PS, called type II reaction [2,11]. The singlet oxygen is extremely reactive and can interact with a large number of biological substrates, inducing oxidative damage and ultimately cell death [11]. The type I reaction can also occur if the excited state of the PS reacts directly with a substrate, such as cell membrane or a molecule, and undergoes hydrogen atom abstraction or electron transfer reactions, to yield free radicals and radical ions. These radicals react with molecular oxygen, producing ROS, such as O_2_^−•^, HO^•^, and H_2_O_2_, which produce oxidative damage that can lead to biological lesions [11]. Figure 2 shows the modified Jablonski diagram of the PDT action mechanism.

The products resulting from type I and type II reactions are responsible for the effect of cell death and therapeutic on PDT. Type I and type II reactions can occur simultaneously and the ratio between these processes depends on the PS, substrate, oxygen concentration, and binding affinity of the sensitizer to the substrate [2,4,13]. However, type II reaction is predominant during PDT, and singlet oxygen is the primary cytotoxic agent responsible for the biological effects [11]. The quantum yield of singlet oxygen formation is one of the most important features of a PS and is determined by the quantum yield and lifetime of its triplet excited state [10]. Due to the high reactivity and short half-life of the ROS, only cells close to the area of the ROS production (areas where the PS is localized) are directly affected by PDT. The extent of damage and cytotoxicity resulting from PDT is multifactorial, depending on the type of PS, its extracellular and intracellular location and the total dose administered, the dose of light (light fluence) and the light fluence rate, availability of oxygen, and the time between PS administration and light exposure [4]. PDT of deeper and hypoxic tumors is more difficult due to the low oxygen concentration and low light penetration into the tissue (light absorption by the PS and energy transfer to the oxygen). On the other hand, more superficial and more oxygenated tumors allow greater production of ROS and thus a more effective PDT treatment [14].

## 3. PDT-Mediated Action Mechanisms

**Figure 3:**
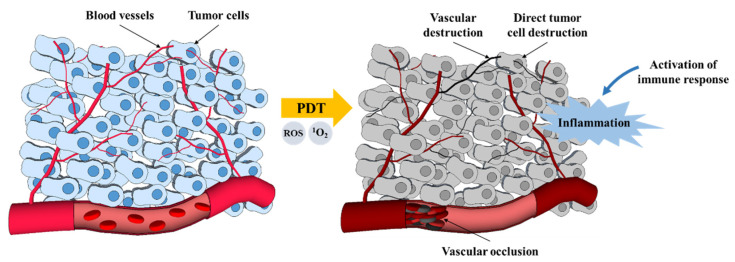
PDT mechanisms for tumor destruction (based on [12]).

There are three main mechanisms by which PDT mediates tumor destruction (Figure 3) [4]. The ROS produced by PDT photochemical reactions can directly destroy tumor cells by inducing apoptosis and necrosis. PDT can also cause the destruction of the tumor-associated vasculature and the surrounding healthy vessels, leading to an interruption of oxygen and nutrient supply and, consequently, to indirect cell death due to hypoxia. Finally, PDT can induce an inflammatory response that activates an immune response against the tumor cells [4,15]. The outcome of PDT depends on all of these mechanisms, and the contribution of each one is determined by the treatment regime used [4,10].

### 3.1. Apoptosis and Necrosis

Tumor destruction from PDT can occur by both programmed (apoptotic) pathways and non-programmed (necrosis) pathways [11,16]. Generally, when high light intensity is employed, the tumor cells are rapidly ablated by necrosis [11]. Necrosis is generally described as a rapid and relatively broad mechanism of cell death, and it is characterized by vacuolization of the cytoplasm and cell membrane breakdown, resulting in a local inflammatory reaction due to the release of cytoplasmic content and pro-inflammatory mediators in the extracellular medium [10].

In contrast, apoptotic death may be initiated by PDT, generally when low light doses are employed [11,16]. Apoptosis is described as a mechanism of programmed cell death that is genetically encoded and energy dependent [10]. Morphologically, it is characterized by chromatin condensation, cleavage of chromosomal DNA into internucleosomal fragments, cell shrinkage, membrane wrinkling, and the formation of apoptotic bodies without plasma membrane breakdown [2,7,10]. No effect or immune response is expected as no toxic chemicals are leaked [11,16].

### 3.2. Vascular Mechanisms

In addition to the direct destruction of tumor cells, the application of PDT often also leads to the destruction of the tumor microvasculature. Like tumor cells, endothelial cells of the vascular system can concentrate PS to create free radicals when activated by appropriate light. The disrupting of the vascular walls leads to the interruption of the tumor’s feeding (i.e., oxygen and nutrients) and, consequently, to the death of the tumor cells. PDT vascular effect has been shown to be very important for the long-term efficacy of PDT [10]. PDT vascular effect can be greatly enhanced by using a short drug–light interval (the time between systemic PS injection and light illumination) when the PS is predominantly localized in the vasculature [1]. Selectivity in vascular PDT protocols is achieved by the precise application of light on the tumor plus a safety margin of the surrounding healthy tissue [10]. Vascular PDT has important advantages in comparison to PDT protocols that require PS accumulation in the tumor cells: it uses photosensitizers that clear rapidly from the organism and minimize skin photosensitivity, gives higher long-term efficacy, and can be performed in one short session [10,17].

### 3.3. Immunological Mechanisms

For many years, PDT was considered a localized treatment, affecting only tumor cells and tumor vasculature. More recently, numerous studies have demonstrated that PDT can significantly influence the adaptive immune response in disparate ways, either through stimulation or suppression of the immune response. The efficacy of PDT appears to be dependent on the induction of antitumor immunity [10]. Long-term tumor control is a combination of the direct effects of PDT on the lesion and its vasculature with upregulation of the immune system [11].

Under certain conditions, PDT induces immunosuppression, which has been mainly associated with reactions to topical treatments with high fluence rates and in large areas of irradiation [10]. In contrast, non-topical PDT treatments are often described as immunostimulatory. The oxidative damage inflicted by PDT on the tumor stroma will eventually result in cell death. When PDT induces necrosis of tumors and their vasculature, an immune cascade is also initiated. The change in tissue integrity and homeostasis triggers an acute inflammatory response initiated by the release of pro-inflammatory mediators, which include various cytokines, growth factors, and proteins [10,11]. These mediators attract the host’s innate immune cells, such as neutrophils, mast cells, macrophages, and dendritic cells, which infiltrate the damaged tissue to restore homeostasis in the affected region [10]. Upon arrival, macrophages phagocytize PDT-damaged cancer cells and present proteins from these tumors to CD4 helper T lymphocytes, which then activate CD8 cytotoxic T lymphocytes [11]. These cytotoxic T cells can recognize and specifically destroy tumor cells and can circulate throughout the body for long periods, ensuring a systemic antitumor immune response [10].

## 4. PDT Essential Elements

### 4.1. Photosensitizers

Photosensitizers are key elements for PDT. Ideally, these molecules should accumulate preferentially in the tumors, have a high singlet oxygen quantum yield, have low activity in the absence of light, be quickly eliminated from the patient body, have amphiphilicity, and have a light absorption peak between approximately 600 nm and 800 nm [18,19].

There are a variety of molecular structures of photosensitizers that are currently used in PDT, and it is possible to divide photosensitizers into three generations. The porfimer sodium and the HpD are first-generation photosensitizers. The second-generation photosensitizers arise to overcome some drawbacks of the first-generation ones, related to light absorption at a specific spectral region. Some examples of second-generation photosensitizers are the derivates of chlorins, bacteriochlorins, and phthalocyanines, which can have a stronger action on the tumor regions due to their strong absorbance in the deep red region, and consequently, increased light penetration. Finally, the third-generation photosensitizers are molecules with improved selectivity for tumor regions, due to the conjunction of the PS with targeting molecules or its encapsulation into carriers. Thus, photosensitizers progressed towards the improvement of PDT specificity and efficacy. Today, the functionalization of photosensitizers seems to be the best strategy to achieve a high selectivity to the tumor regions, combining photosensitizers with biomolecules or carriers [18,19,20]. Photosensitizers can be covalently bonded to several biomolecules, which have affinity to tumors. These biomolecules include antibodies, proteins, carbohydrates, and others. Photosensitizers can also be encapsulated into carriers, such as gold nanoparticles, silica nanoparticles, quantum dots, carbon nanotubes, or others carriers, to guide the photosensitizers to tumors [19,21,22,23,24].

Table 1 presents the most used photosensitizers in clinical PDT, including their trade name and class, molecular formula, excitation wavelength, quantum yield, extinction coefficient, and main applications [10,18,25,26,27,28,29,30,31]. A variety of photosensitizers under clinical trials for approval in clinical PDT are presented in Table 2 [10,18,26,27,32,33,34].

Consensus protocol for conventional topical PDT recommends some lesion preparation to increase PS absorption and light penetration. The typical PDT topical treatment protocol follows the next steps [35]:

- Wash the area to be treated with soap and water;
- Remove any residue and remaining oil with a gauze soaked in acetone or alcohol;
- Apply the PS evenly over the entire area to be treated. Apply a second layer of PS after the first one has dried;
- Allow the PS to incubate for 0.5–4 h;
- Activate the PS with the appropriate light source;
- Wash the treated area with soap and water to remove any residual PS;
- Avoid any direct sunlight for 48 h;
- Repeat as needed in 2–3 weeks.

In dermatological indications, PDT is usually performed by topical application of PS, in particular 5-aminolevulinic acid (5-ALA) or its ester methyl aminolevulinate (MAL) [35]. Three photosensitizers are currently approved for topical use in Europe: MAL Metvix^®^, 5-ALA Ameluz^®^, and 5-ALA AlaCare^®^. MAL Metvix^®^ is used along with red light to treat actinic keratosis, Bowen’s disease, and superficial basal cell carcinoma (3 h of drug-light interval and 37–75 J/cm^2^ of total light dose). 5-ALA Ameluz^®^ is used in combination with red light for the treatment of mild and moderate actinic keratosis and superficial basal cell carcinoma (3 h of drug-light interval and 37–200 J/cm^2^ of total light dose). MAL Metvix^®^ and 5-ALA Ameluz^®^ are also used with daylight to treat moderate actinic keratosis (0.5 h of drug-light interval and exposure during 2 h). 5-ALA AlaCare^®^ is approved for the treatment of mild actinic keratosis with red light (4 h of drug-light interval and 37 J/cm^2^ of total light dose). A 20% formulation of 5-ALA Levulan^®^ is approved in North America for the treatment of actinic keratosis with blue light (14–18 h of drug-light interval and 10 J/cm^2^ of total light dose). Besides, topical PDT is highly recommended for the photorejuvenation and the treatment of acne vulgaris, although these indications currently lack approval for use and the protocols still need to be optimized [35,36,37,38].

**Figure 4:**
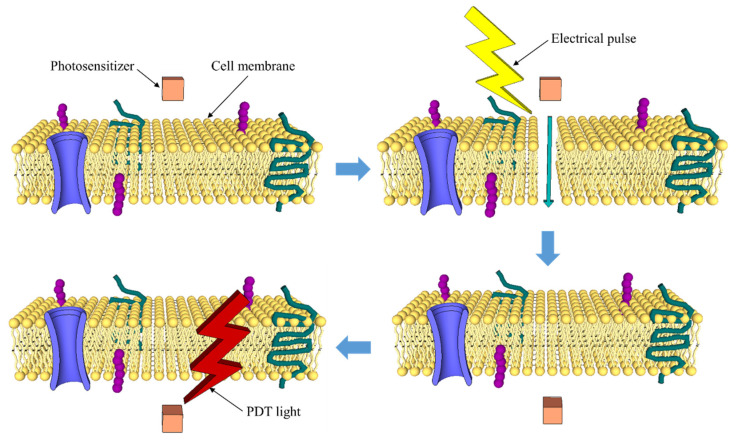
PDT with electroporation (based on [20]).

A strategy that seems to be of great interest in the near future is to increase the PDT selectivity through the development of activatable multifunctional photosensitizers, which become active after receiving a biological or physical stimulus. Biological stimuli include the physiological conditions associated with cancer, such as temperature, pH, and enzymatic activity. For example, the peptidic zipper-based PS was designed to react under acidic conditions. Physical stimuli refer to an artificial agent activation, applying magnetic or electric fields, ultrasounds, two-photon excitation, etc. [18,19]. For example, electroporation can be used to support PDT (Figure 4). Electroporation is reported as an effective method that could be used to increase the transport of a PS into the pathological cells, which could lead to the increase of cytotoxicity and PDT efficacy. Several studies have been performed over the years, using different photosensitizers, the clinically approved Photofrin PS being the most relevant. The results, including a study performed with human cancer cells, conclude, undoubtedly, that PDT with electroporation is an attractive approach to cancer treatment, but detailed studies on the mechanisms of this approach are still required [20,39].

Very recently, the possibility of using transition metal coordination complexes or organic fluorophores as efficient photosensitizers for PDT has also been reported. The transition metal coordinator complexes, such as ruthenium(II) complexes, iridium(III) complexes, and polymetallic complexes, meet several basic needs for PDT. The most relevant feature is their heavy-atom effect, which mediates strong spin–orbital coupling, providing more time for the excited states to interact with molecular oxygen. Between other features, it can be also be stated that they are easily synthetized, including the possibility of fine-tuning their properties. The organic fluorophores, such as naphthalimides, xanthenes, boron dipyrromethene (BODIPY), and cyanines, can also be designed as photosensitizers for cancer PDT, with high light absorption at relatively long wavelengths and large molar extinction coefficient. Organic fluorophore photosensitizers have low toxicity, good biocompatibility, and long triplet lifetimes, and their fluorescence emission can be used to perform real-time monitoring during PDT treatment [40,41].

### 4.2. Light

PDT has been performed with various light sources, including lasers, incandescent light, and laser-emitting diodes [42]. Laser light sources are usually expensive and require the use of an optical system to expand the light beam for irradiation of a larger tissue area. Non-laser light sources (e.g., conventional lamps) can be used with optical fibers to specify the light wavelength for tissue irradiation. However, conventional lamps may have thermal effects, which must be avoided in PDT. Finally, light-emitting diodes (LEDs) have also been used in PDT as light sources. LEDs are less expensive, less hazardous, thermally non-destructive, and easily available in flexible arrays [43]. Light penetration into tumor tissue is very complex, as it can be reflected, scattered, or absorbed. The extent of these processes depends on the type of tissue and the wavelength of light [44]. Light absorption is mainly due to endogenous chromophores existing in tissues, such as hemoglobin, myoglobin, and cytochromes, which can reduce the photodynamic process by competing with PS in the absorption process [44,45]. Light absorption by tissues decreases with increasing wavelength, so longer wavelengths of light (red light) penetrate more efficiently through tissue. The region between 600 and 1200 nm is often called the “tissue optical window” [12,44]. Shorter wavelengths (<600 nm) have less tissue penetration and are more absorbed, resulting in increased skin photosensitivity. On the other hand, longer wavelengths (>850 nm) do not have enough energy to excite oxygen in its state of singlet and to produce enough reactive oxygen species. Therefore, the highest tissue permeability occurs between 600 and 850 nm. This range, called the “phototherapeutic window,” is predominantly used in PDT [10,12,18,20].

As light is an essential component of PDT, clinical efficacy is highly dependent on the accuracy of its delivery to the target tissue and its dose, which translates into light fluence, light fluence rate, light exposure time, and light delivery mode (single or fractionated) [12]. Light fluence is the total energy of exposed light across a sectional area of irradiated spot and is expressed in J/cm^2^. Light fluence rate is the incident energy per second across a sectional area of the irradiated spot and is expressed as W/cm^2^ [4,46,47].

Several studies have reported that low light fluence rates are advantageous for PDT [48,49,50]. The main reason for the lower efficacy of PDT for high light fluence rates is the oxygen depletion in tissues, which leads to a low photo-degradation of the PS. The light fluence rate has also been shown to have an impact on the dominant mechanism of cell death in the PDT. The use of low light fluence rates increases the selective apoptosis of tumor cells, which is more desirable than inflammation and edema that usually occur with the uncontrolled rupturing of cellular content in necrosis [51].

Another relevant light source for PDT is the natural light. The concept of daylight PDT is based on the use of natural light instead of an artificial light source to treat skin lesions, such as actinic keratosis. The main advantages of using daylight PDT are minimal patient discomfort and shorter clinical visits (patients can complete their therapy at home). Moreover, daylight PDT seems to be as effective as conventional PDT for actinic keratosis. For this specific application, the patients expose the sites to daylight for 2 h, after 30 min of PS application. The short PS incubation time in the daylight PDT, compared with conventional PDT (1–3 h required), allows a smaller and more continuous PS activation, leading to lower patient pain intensity associated with PDT [52,53].

### 4.3. Oxygen

The third key component in the PDT mechanism is molecular oxygen. Oxygen is crucial for the production of ROS during PDT. Oxygen concentration in the tissues truly affects the effectiveness of the PDT treatment. In fact, oxygen concentration can vary significantly between different tumors and even between different regions of the same tumor, depending on the density of the vasculature. Especially in deeper solid tumors, often characterized by their anoxic microenvironment, lack of oxygen can be a limiting factor. As mentioned above, the irradiation of the tumor with a high light fluence rate can lead to a temporary local oxygen depletion. This leads to interruption of ROS production and reduced treatment effectiveness. Oxygen depletion occurs when the rate of oxygen consumption by the photodynamic reaction is greater than the rate of oxygen diffusion in the irradiated area. In addition, PDT can cause occlusion of the tumor vasculature, reducing blood flow to the tumor tissue, further increasing hypoxia [10,26,46].

Real-time measurement of the tissue oxygen levels, before and during PDT, is one of the main challenges in the near future. This allows to optimize the PDT therapeutic result by adjusting the light fluence rate (increasing the irradiation time to maintain the total light dose) or using fractional light dose. Several sensors have been used to monitor the oxygen level in biological media, using imaging agents. However, the combination of these imaging agents with PS has been hardly reported [18]. Other methods to increase oxygen availability in the tumor have been tested: indirect introduction of oxygen and direct introduction of oxygen. One indirect way to increase oxygen concentration in tumor cells is using catalase enzyme to decompose the intracellular hydrogen peroxide into oxygen. The direct delivery of oxygen into tumors is achieved by using oxygen carriers, such as perfluorocarbons and hemoglobin, commonly used to overcome tumor hypoxia in the PDT procedure [37].

## 5. Advantages and Limitations of PDT

PDT has several advantages over conventional approaches to cancer treatment. First-generation photosensitizers cause increased skin photosensitivity. However, PDT has no long-term side effects when correctly used. It is less invasive than surgical procedures and can be performed on an outpatient basis. In addition to the tumor itself, PDT can also destroy the vasculature associated with it, greatly contributing to tumor death [54]. PDT can be applied directly and accurately in the target tissue, due to its dual selectivity. The two main factors that contribute to the selectivity of PDT are the intrinsic capacity of some photosensitizers to preferentially accumulate in tumor tissue and light irradiation exclusively in the target tissue [10,54]. The selective accumulation of the PS in the tumor is facilitated in the case of topical application, since PS is applied directly and only to the lesions to be treated. When PS is given intravenously, it needs to remain in circulation long enough to reach and accumulate in the tumor [10]. Furthermore, PDT can be repeated several times in the same location, unlike radiation. There is little or no scarring after healing. Finally, it usually costs less than other therapeutic modalities in cancer treatment [54,55].

Like every therapeutic modalities, PDT also has some limitations. The photodynamic effect occurs selectively in the irradiated site, which makes its use in disseminated metastases very difficult with the currently available technology [54]. Tissue oxygenation is crucial for the photodynamic effect to occur, so tumors surrounded by necrotic tissue or dense tumor masses can lead to ineffective PDT. Finally, the accuracy of target tissue irradiation is the most important point when considering PDT as a treatment option. Therefore, deep tumors (not easily accessible without surgical intervention) are difficult to treat due to the low penetration of visible light into the tissue [54,56]. The main advantages and limitations of PDT are summarized in Table 3.

## 6. Applications of PDT

PDT is a minimally invasive procedure that is clinically used in the treatment of several oncologic human diseases, such as skin, esophageal, head and neck, lung, and bladder cancers [57]. However, PDT also has several non-oncologic applications [58], including the treatment of non-cancerous human diseases, such as dermatologic (acne [59], warts [60], photoaging [61], psoriasis [62], vascular malformations [63], hirsutism [64], keloid [50], and alopecia areata [65]), ophthalmologic (central serous chorioretinopathy [66] and corneal neovascularization [67]), cardiovascular (atherosclerosis [68] and esophageal varix [69]), dental (oral lichen planus [70]), neurologic (Alzheimer’s disease [71]), skeletal (rheumatoid arthritis [72]), and gastrointestinal (Crohn’s disease [73]).

An extension of PDT procedure can be the inactivation of viruses and microorganisms, including bacteria, yeasts, and fungi, named as photodynamic inactivation of microorganisms (PDI). The viruses or microorganisms are inactivated by combining non-toxic dyes (photosensitizers) with harmless visible light. PDI can be used as an alternative to the use of antibiotics and antiviral drugs that usually cause resistance. The application of PDI is possible in several areas, including human and veterinary medicine, agro-food, wastewater treatment, and biosafety. However, PDI in the treatment of infections is easier to perform in vitro, compared with its clinical applicability, due to the low tissue penetration depth of visible light. Light applied intravenously can be a solution during the clinical treatment of infections. Very recently, the use of the PDT procedure to treat patients with COVID-19 has also been discussed [8,57,74,75,76,77,78,79,80,81].

Dr. M. Weber et al. [8] performed a study to evaluate if the PDT procedure with Riboflavin (also known as vitamin B2) and blue light can be used effectively as a therapy for patients infected with acute COVID-19. The study used COVID-19-positive patients who received PDT therapy and COVID-19-positive patients who received conventional care. The patients that received PDT treatment showed significant improvement in clinical symptoms and viral load within 5 days. The control patients had no significant improvement in clinical symptoms or viral load within 5 days. The results prove the potential of PDT procedure to treat patients infected with COVID-19 virus at an early infection stage and with mild to moderate clinical symptoms. This new application of PDT procedure can prevent hospitalization and intensive care treatments.

**Figure 5:**
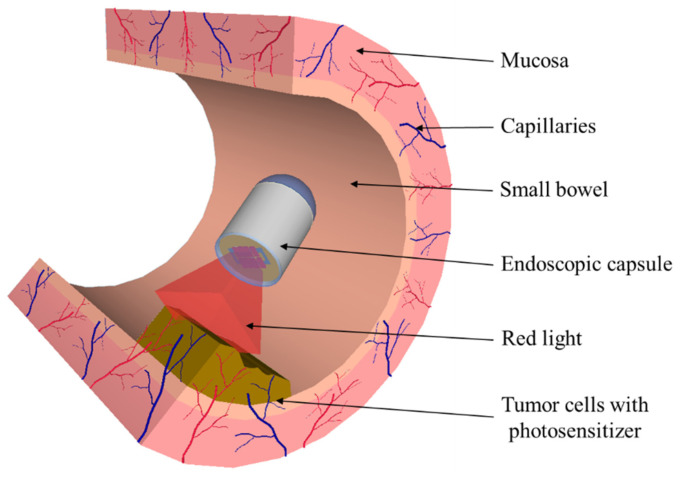
Illustration of PDT integrated in the endoscopic capsule (Adapted with permission from [83], IEEE, 2019).

Finally, PDT can be implemented in a medical device, e.g., endoscopic capsule. In 2016, G. Tortora et al. [82] developed an ingestible capsule for light delivery in PDT treatment of *Helicobacter pylori* infection. *Helicobacter pylori* bacterium is known to be photosensitive without the exogenous assumption of photosensitizers. This bacterium can be killed by exciting the photosensitizers naturally present in it with the appropriate light wavelength. The capsule with 27 mm length and 14 mm diameter has been equipped with 8 LEDs positioned on an electronic board with a magnetic switch (to turn on the capsule’s power) and a battery. The capsule light-emitting module was dimensioned considering the required light necessary to kill the bacterium, with blue (405 nm) and red (625 nm) lights. In 2018, J. A. Rodrigues et al. [26,83] studied the photodynamic activity of the mTHPC (Foscan^®^) on RKO and HCT-15 cell lines to implement PDT in autonomous medical devices, such as endoscopic capsules for clinical treatment of several gastrointestinal tract tumors. Figure 5 envisages the integration of PDT in the endoscopic capsule. Due to the endoscopic capsule dimensions and battery life, the light fluence and fluence rate of the red light must be minimized to reduce the PDT treatment time. The experimental results showed that a small amount of mTHPC (0.15 mg/kg) and light fluence (5–20 J/cm^2^) is sufficient to obtain significant photodynamic activity. An array of LEDs with peak transmittance at 652 nm was used in the in vitro PDT assays. The experimental results show that decreased cell viability (down to 30%) can be obtained for 1–5 μg/mL of mTHPC concentrations and 2.5 J/cm^2^ of light fluence. The use of a minimum light fluence (2.5 J/cm^2^) and light fluence rate (11 mW/cm^2^) allowed to reduce the treatment time to just 3 min and 47 s. The PDT endoscopic capsule was designed with two functional sides. The round side is compound of the conventional optical system of the endoscopic capsule, consisting of the CMOS image sensor, four white LEDs, and focal lenses, and the planar side constitutes the therapeutic module, composed of a red light source (array of 8 SMD red LEDs with total fluence rate of 14 mW/cm^2^) and a magnetic switch for turning the red light on and off. This capsule has magnetic locomotion control, for immobilization of the capsule during the treatment time, and is 31 mm in length and 14 mm in diameter. mTHPC-mediated PDT, using a light fluence of 2.5 J/cm^2^ and fluence rate of 14 mW/cm^2^, reduces the PDT treatment time to approximately 3 min. Faster treatment requires less battery capacity and therefore fewer capsules.

## 7. Conclusions

PDT is one of the most interesting and promising approaches to treat various oncologic, non-oncologic, and infectious diseases. This review presented the main principles, mechanisms, and crucial elements of PDT. PDT is based on the dynamic interaction between a PS, light with a specific wavelength, and molecular oxygen, to produce ROS that promote selective destruction of the target tissue. The evolution of photosensitizers was also addressed in this paper, including their future trends. Photosensitizers are evolving towards increasing the PDT efficacy and selectivity, and many possibilities are currently under research. One strategy to increase PDT selectivity involves the development of multifunctional photosensitizers that can be activated by a biological or physical stimulus.

PDT has been increasingly used in many applications, such as destroying tumor tissues, bacteria, fungi, and viruses (including COVID-19). Moreover, PDT can be integrated in medical devices. A light delivery capsule has been developed for mTHPC-mediated PDT treatment of several gastrointestinal tract tumors. The good photodynamic response at low light fluence and low light fluence rate allows to reduce the treatment time to a few minutes and thus integrate the PDT in autonomous medical devices, such as endoscopic capsules of very small dimensions, to provide them with an advanced therapeutic function.

The interdisciplinary uniqueness of PDT inspires physicists, chemists, biologists, and physicians, and its further development and discovery of new applications will only be limited by their enormous imagination.

## Figures and Tables

**Table 1:** Photosensitizers used in clinical PDT.

Trade Name (Class)	Molecular Formula	Excitation Wavelength (nm)	Quantum Yield	Molar Extinction Coefficient (M^−1^ cm^−1^)	Main Applications
Photofrin^®^ (porphyrin)	C_34_H_38_N_4_NaO_5_^+^	630	0.01 in PBS	3.0 × 10^3^ in PBS	Esophageal, lung, and endobronchial cancers
Ameluz^®^ (porphyrin)	C_5_H_9_NO_3_^•^HCl	630	-	-	Actinic keratosis and basal cell carcinoma
AlaCare^®^ (porphyrin)	C_5_H_9_NO_3_	630	-	-	Actinic keratosis
Levulan^®^ (porphyrin)	C_5_H_9_NO_3_	635	0.56	5.0 × 10^3^	Actinic keratosis
Hexvix^®^ (porphyrin)	C_11_H_21_NO_3_	635	-	<1.0 × 10^3^	Bladder cancer
Foscan^®^ (chlorine)	C_44_H_32_O_4_N_4_	652	0.43 in methanol	3.0 × 10^4^ in methanol	Head and neck cancers
Laserphyrin^®^ (chlorine)	C_38_H_37_N_5_O_9_	664	0.77 in PBS	4.0 × 10^4^ in PBS	Lung and esophageal cancers and brain tumors
Metvix^®^ (porphyrin)	C_6_H_11_NO_3_	570–670	-	<1.0 × 10^3^	Basal cell carcinoma, Bowen’s disease, and actinic keratosis
Visudyne^®^ (porphyrin)	C_82_H_84_N_8_O_16_	690	0.7 in methanol	3.4 × 10^4^ in methanol	Age-related macular degeneration
Redaporphine^®^ (LUZ11) (bacteriochlorin)	C_48_H_38_F_8_N_8_O_8_S_4_	749	0.43 in ethanol	140 × 10^3^ in ethanol	Biliary tract cancer

**Table 2:** Photosensitizers under clinical trials for use in PDT.

Trade Name	Molecular Formula	Excitation Wavelength (nm)	Quantum Yield	Molar Extinction Coefficient (M^−1^ cm^−1^)	Main Applications
Radachlorin^®^ (chlorine)	C_34_H_36_N_4_O_6_ C_33_H_34_N_4_O_5_ C_33_H_34_N_4_O_6_	662	0.52–0.62	3.42 × 10^4^	Skin cancer
Photochlor^®^ (chlorins)	C_39_H_48_N_4_O_4_	664	0.48 in CH_2_Cl_2_	4.75 × 10^4^ in 1% Tween-80 micelles	Head and neck cancer
Purlytin^®^ (purpurin)	C_37_H_42_Cl_2_N_4_O_2_Sn	664	0.7 in acetonitrile	2.8 × 10^4^	Age-related macular degeneration
Fotolon^®^ (chlorin)	C_34_H_36_N_4_O_6_	665	0.63 in dimethylformamide	5.0 × 10^4^ in diethyl ether	Nasopharyngeal sarcoma
Lutrin^®^ (texaphyrin)	C_52_H_72_LuN_5_O_14_	732	4.2 × 10^4^ in methanol	0.11 in methanol	Coronary artery disease
TOOKAD^®^ (WST09) (bacteriochlorin)	C_37_H_41_K_2_N_5_O_9_PdS	762	0.99 in organic solvent	8.85 × 10^4^	Prostate cancer

**Table 3:** Summary of the main advantages and disadvantages of PDT.

Advantages	Limitations
☑ Low side effects ☑ Less invasive ☑ Short treatment time ☑ Usable in outpatient basis ☑ Cancer selectivity ☑ Multiple applications at the same location ☑ Excellent cosmetic outcome ☑ Lower costs	⊠ Photosensitivity after treatment ⊠ Treatment efficacy dependent on accuracy of tumor light irradiation ⊠ Tissue oxygenation is crucial for the photodynamic effect ⊠ Very difficult to treat metastatic cancers with current technology

## Data Availability

Not applicable.
